# Supplementary figures and images for: Splice site m6A methylation prevents binding of DGCR8 to suppress KRT4 pre-mRNA splicing in oral squamous cell carcinoma
Source: PeerJ. 2023 Feb 16;11:e14824. doi: 10.7717/peerj.14824 (PMC9939020; doi:10.7717/peerj.14824)

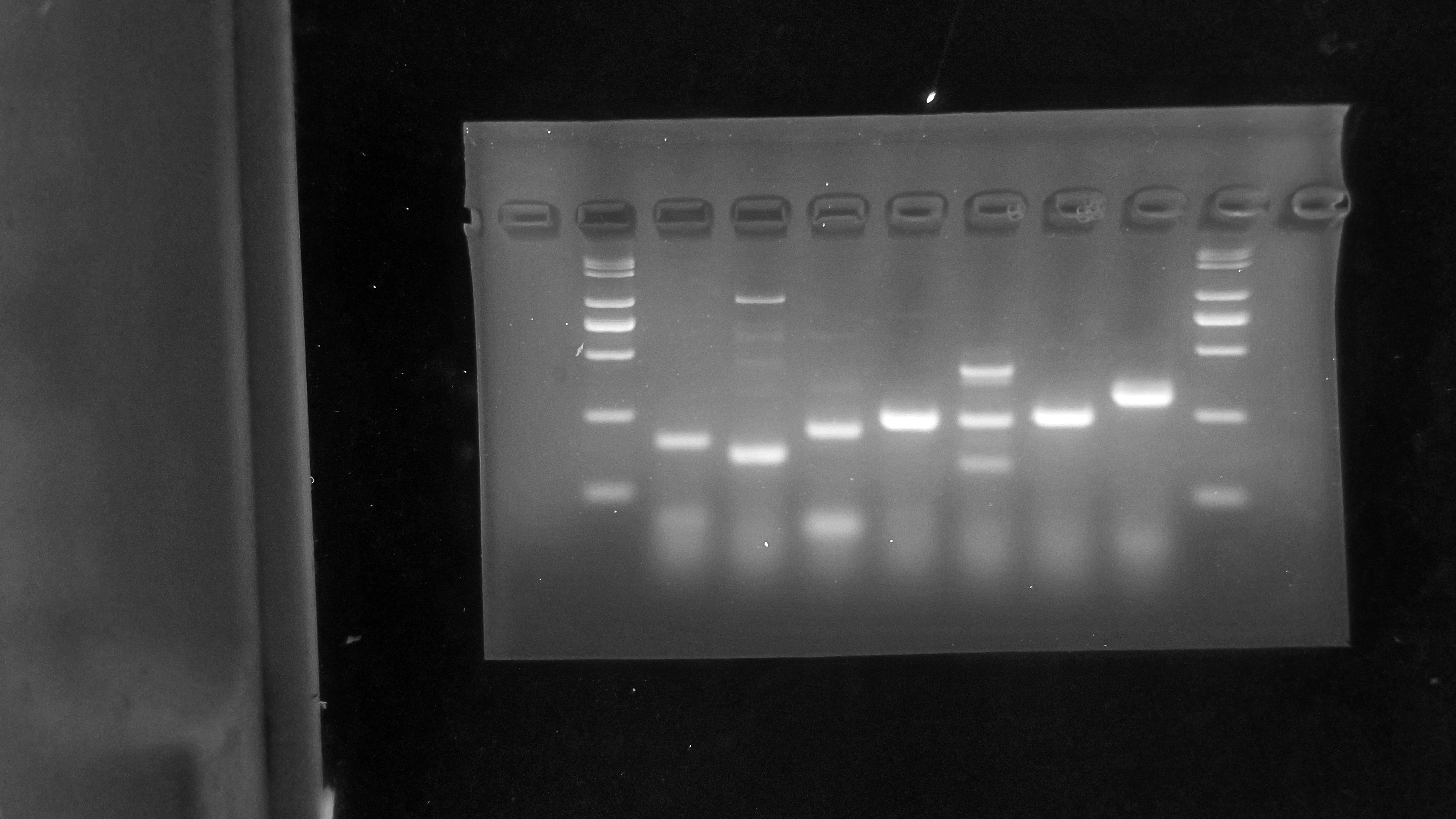

Supplement: Supplemental Information 1 [file peerj-11-14824-s001.zip › Raw data/Figure 1/Fig 1B HN6 cell.jpg]

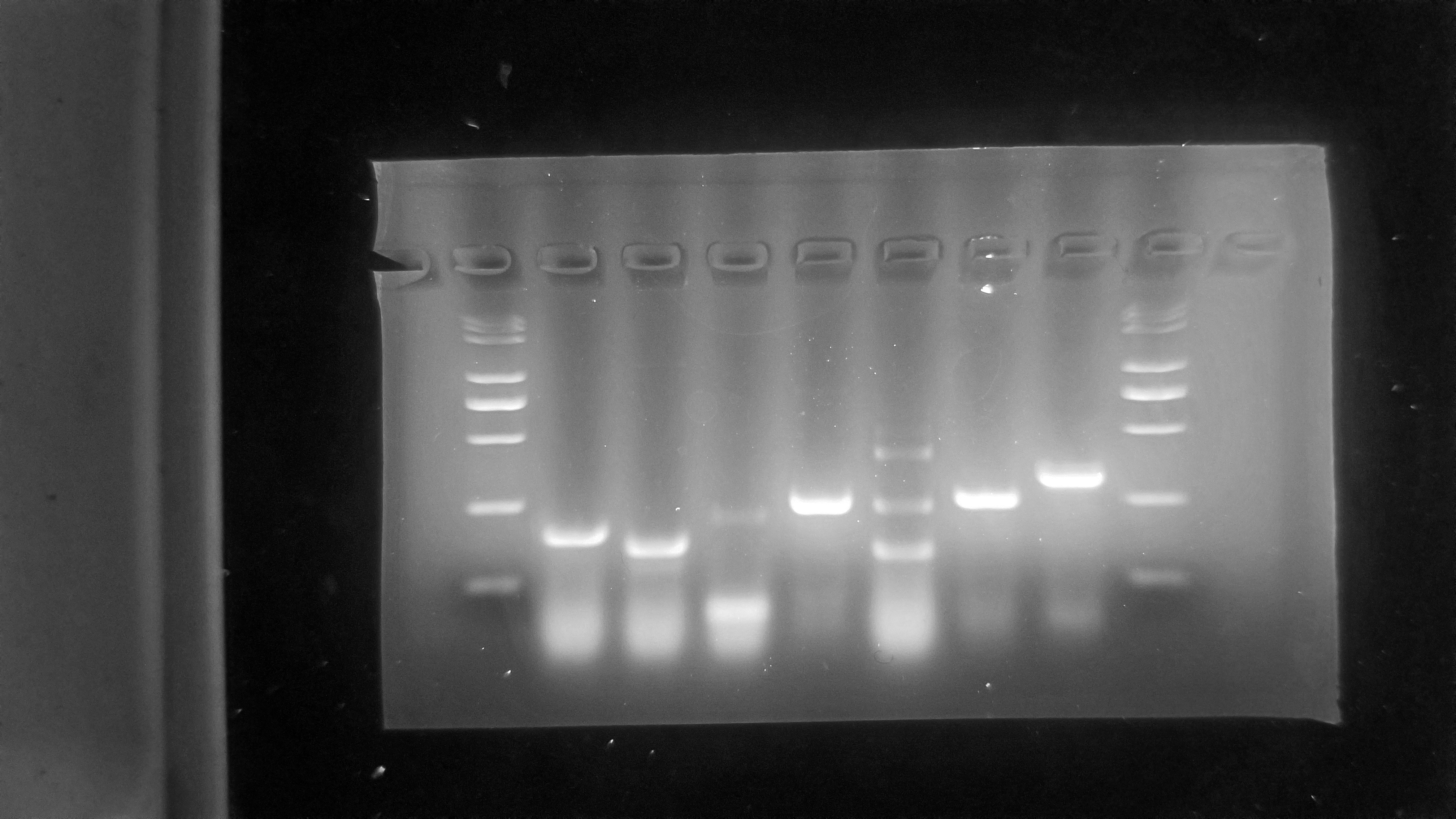

Supplement: Supplemental Information 1 [file peerj-11-14824-s001.zip › Raw data/Figure 1/Fig 1B NOK cell.jpg]

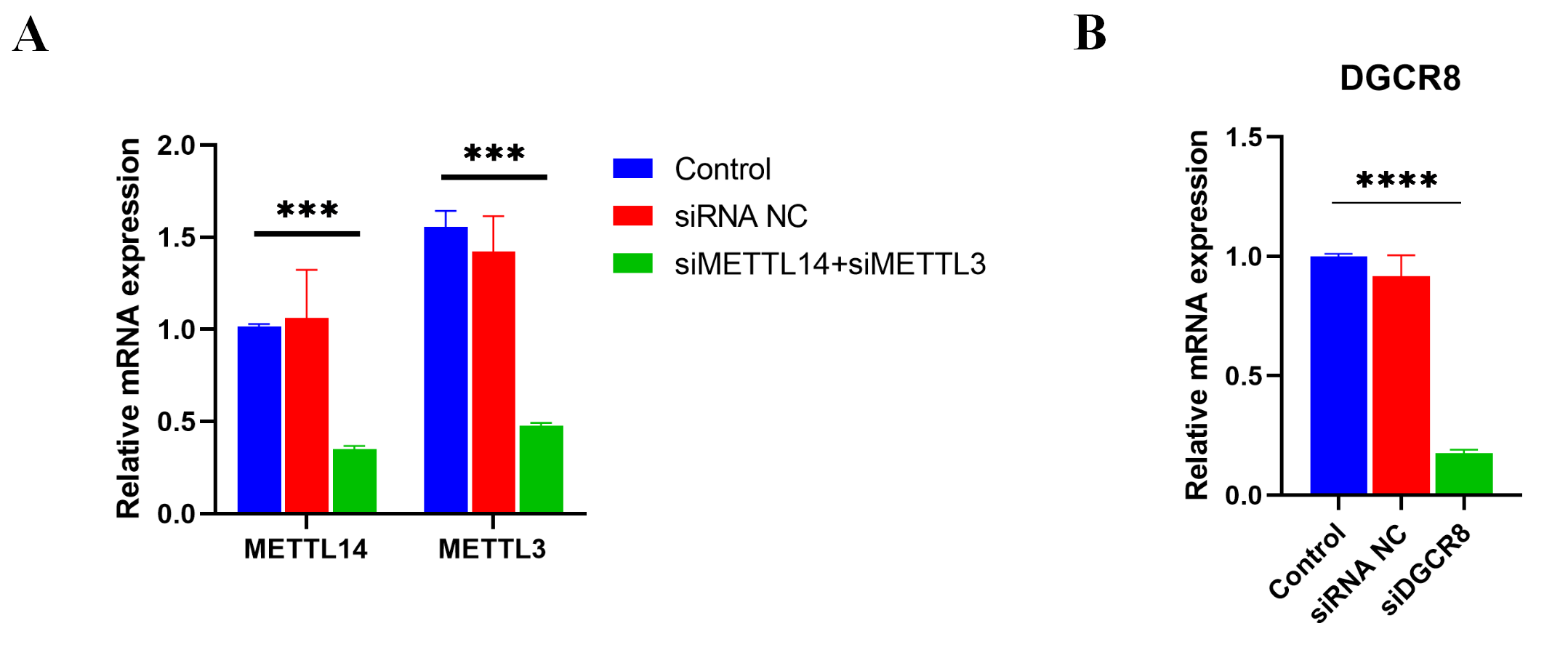

Supplement: Supplemental Information 2 — The level of METTL3 (A), METTL14 (A) and DGCR8 (B) detected by qRT-PCR in HN6 cells treated with or without siRNAs. NC, negative control; siMETTL3, METTL3 siRNA; siMETTL14, METTL14 siRNA; siDGCR8, DGCR8 siRNA. ∗∗∗P < 0.001, ∗∗∗∗P < 0.0001. [file peerj-11-14824-s002.png]
